# Health professionals’ experience and perceived obstacles with managing patients’ medication information in Norway: cross-sectional survey

**DOI:** 10.1186/s12913-023-10485-9

**Published:** 2024-01-13

**Authors:** Bo Wang, Unn Sollid Manskow

**Affiliations:** 1 Norwegian Centre for E-Health Research, University Hospital of North Norway, Sykehusvegen 23, Forskningsparken, Tromsø, Norway; 2 Department of Health and Care Sciences, Centre for Care Research, UiT- The Arctic University of Norway, Tromsø, Norway

**Keywords:** Medication information, Medication reconciliation, e-medicines management, Shared Medication Lists, Health professionals, Polypharmacy, Experiences, Obstacles

## Abstract

**Background:**

Access to correct and up to date medication information is crucial for effective patient treatment. However, persistent discrepancies exist. This study examines the experiences and challenges health professionals encounter while utilizing current digital solutions in the Norwegian healthcare system to manage patients' medication information.

**Methods:**

A cross-sectional descriptive analysis using quantitative survey data was conducted to investigate how health professionals managed patients’ medication information. Content analysis was used to analyze free-text responses concerning challenges they encountered when transferring medication information and to identify factors deemed necessary for implementing the Shared Medication List in Norway.

**Results:**

A total of 262 doctors and 244 nurses responded to the survey. A higher percentage of doctors (72.2%) expressed concerns regarding obtaining accurate and updated medication lists than nurses (42.9%), particularly for patients with polypharmacy (35.3%) or transitioning between primary and specialist care services (27.6%). The patient's verbal information was the main source for hospital doctors (17%) to obtain an overview of the patient’s medication usage, while general practitioners (19%) and nurses (working in both primary and specialist care services, 28% and 27% respectively) predominantly relied on electronic prescriptions. Doctors, in particular general practitioners, reported carrying excessive responsibilities in coordinating with other health actors (84.8%) and managing patients' medication information. The vast majority of both doctors (84.4%) and nurses (82.0%) were in favor of a Shared Medication List. However, about a third of doctors (36.3%) and nurses (29.8%) expressed the need for a more balanced responsibility in updating and managing patients' medication information, while ensuring compatibility with existing digital systems.

**Conclusions:**

Fragmented resources for medication information and unclear responsibilities were prevalent concerns among both professional groups. Doctors voiced more concern than nurses about the accuracy of patients’ medication list. While both groups are positive about a shared medication list, successful implementation requires proactive training initiatives and clearer role clarification.

**Supplementary Information:**

The online version contains supplementary material available at 10.1186/s12913-023-10485-9.

## Background

Unsafe medication management, medication errors and other drug related problems are widely recognized as leading causes of preventable harm in health systems worldwide, resulting in a staggering annual cost of over €40 billion globally [1]. Recent studies indicated that drug-related incidents account for 2.3–28.6% of admissions to emergency departments with elderly patients on polypharmacy being most affected [2–5]. Besides polypharmacy patients, the transitions of care emerges as a critical risk point for medication errors due to inadequate transfer of information about prescribed medication among health professionals [1]. The access to accurate and up to date information about a patient’s medicine list is of utmost importance in both prevention and treatment of medical conditions [6]. Unfortunately, this crucial information is often not available due to inadequate integration among existing digital systems across healthcare services, thereby creating the potential for errors, adverse events, and even premature deaths [6–8]. Furthermore, the process of obtaining accurate information and reconciling medication lists remains exceedingly time-consuming and complex, involving manual procedures that threaten both medication safety and the overall quality of care [9, 10].

Health information exchange serves as a critical avenue for the digital exchange of information across various healthcare organizations and levels of care, with the overarching aim of enhancing patient safety. However, previous studies showed that fragmented information systems engender poor communication and disrupt the flow of information, leading to potentially harmful medication errors [11]. Reported barriers regarding medication information exchange is incomplete information, inefficient workflow, and the exchanged information not meeting the needs of the user. In addition, potential facilitators are identified as obtaining more patient information, a thoughtful workflow folding in health information exchange, and strong user involvement early in the implementation process [12, 13]. Interaction and communication through seamless digital information systems across levels of care, as well as clarified responsibilities between all actors involved are main factors for improving the quality and accuracy of patients’ medication lists [9]. This also includes patients themselves, along with their caregivers, as they are important sources of medicine information and can potentially reduce the workload for health professionals and increase quality of the information [14, 15].

The adoption of digital systems designed for sharing medication information emerges as the key strategy to mitigate the vast number of medication errors prevalent within the Norwegian healthcare services [16]. Currently, one such system—the Shared Medication List (SML) – is piloting in one health region in Norway, with national implementation set to commence in 2024 [17]. The SML is a centralized, real-time, and up-to-date medication records extending across healthcare services and levels. In practice, instead of local medication lists in individual physician’s electronic health record (EHR) systems, physicians now have access to a national database, integrating the SML with their EHRs. Any physician who prescribes medications can access and edit the SML. Other health professionals (i.e., nurses) can view, but not edit, the SML through the Summary Care Record (SCR) interface. Its introduction aims to enhance patient safety and quality of care by decreasing time and resource health professionals spent reconciling medication lists, simplifying work processes, and preventing medication errors during care transitions [17]. However, while digital interventions of this nature hold potential to improve effectiveness, efficiency, accessibility, and safety in healthcare delivery, the existing body of scientific evidence pertaining to the positive effects of digitally shared medication lists is limited [18–20]. Equally crucial is investigating the implementation process, especially considering past initiatives faced by instances of non-adoption or unsuccessful attempts at scale up locally, spread distantly, and sustained long-term viability [21, 22].

This article is part of a pre-study in an ongoing mixed-method study during 2019–2025 investigating the effects and experiences of end-users (health professionals and patients) *before*, *during* and *after* the implementation of the SML. The overall objective is to produce research-based knowledge on the introduction of the SML in the Norwegian healthcare system, focusing on access to medication information, medication safety, efficiency, work processes and interprofessional collaboration. The main study will provide transferrable knowledge relevant for decision makers about the impact and conditions of the SML within and across health care organizations and end-users as a key tool for the health authorities responsible for implementing the SML. The study consists of three sub-studies: 1) pre-study (prior to implementation), 2) early evaluation (during), and 3) after the implementation of the SML. Previously, the qualitative part of this pre-study has been published investigating in-dept knowledge of health professionals' experiences with obtaining and exchanging correct information about patients' medication list in a selection of municipalities in Norway [9].

This study aimed to investigate how Norwegian health professionals experience access to and the exchange of patient medication information within the context of the current digital systems. In response, we presented the findings on the prevalence of experiences among a larger sample of doctors and nurses, addressing the following research questions:

i) Are there differences in the experiences of doctors and nurses when managing patients’ medication information?
ii) Are there differences in the obstacles encountered by doctors and nurses when managing patients’ medication information?
iii) What factors do doctors and nurses deem important for the successful implementation of SML?

## Methods

The cross-sectional survey was conducted in September–October 2022. The STROBE guidelines (see Supplementary 1) for observational studies were consulted during the design and reporting of this cross-sectional study [23].

### Survey

The survey (available at Supplementary 2), developed in collaboration with Linné University in Sweden between 2020–2021, was designed to collect baseline data on health professionals’ experiences accessing patients’ medication information in current digital systems. This data will facilitate tracking changes during and after the implementation of SML, as well as enable comparisons across regions and countries. A future comparative study between Norway and Sweden is planned.

A subset of ten questions from this survey has already been reported in a white paper report [24] for a Swedish population, encompassing both health professionals and patients. The questions were developed based on previous qualitative interviews in Norway [9] and Sweden [24], previous research and feedback from the projects reference group in both countries. The questions are validated through cognitive interviews for the different health professional groups, pilot test of the survey and feedback from other researchers with expertise within survey construction. Pilot testing of the survey has been done both in Norway and Sweden within different health professional groups including doctors, nurses, and pharmacists. The survey was distributed by Medlytics^1^ to health professionals both in the municipal and specialist health care in Norway.

The survey included a total of 32 questions (see Supplementary 2), comprising three sections. Background information (8 questions) including age group, gender, profession (doctor, nurse, other), years of professional experience (0–5, 6–10, 11–15, 16–20, 21–25, > 25y), region/county, size of municipality (large, medium, small), and primary workplace (hospital, nursing home, home care services, general practice, emergency clinic, other). An additional open-ended question inquired about the EHR system used by the respondents (see supplement 3, 4). Information source and frequency (9 questions): Participants were asked about the type of information sources they used and the frequency of their utilization (never, sometimes a year, sometimes per month, sometimes per week, daily or not relevant*).* The Managing patients’ medication information (13 questions) section explored health professionals’ experiences and obstacles with handling information about patients' medications. Using a 6-point Likert scale (1-completely disagree, 6-completely agree), participants rated their agreement with a mix of positive and negative statements.

In addition, the survey contained two free-text questions asked 1) are there patient groups, situations, or transmissions where information about patients’ medication work works less well today? 2) Do you see any obstacles or problems related to the introduction of the shared medication list?

### The sample

In the planning phase of the survey, we determined that a minimum of 500 respondents would be adequate to address our research questions. The target sample includes primarily doctors, nurses, and pharmacists, but also other health personnels, in hospital and municipal settings. We presented the selection criteria in the current study as follows.

### Inclusion criteria

The respondents were selected based on predetermined inclusion criteria, which specified certain professions and workplaces. The eligible professions consisted of doctors, nurses/nurse specialists, and social educators/social educator specialists. In the Norwegian healthcare context, the term "*social educators*" refers to nurses with specialized training in caring for individuals with developmental disabilities. Hence, nurses/nurse specialists and social educators/social educator specialists were grouped together under the category of nurses in this study.

The eligible workplaces were specialist health services (public hospitals), and municipal health services such as general practitioners (GP), nursing homes, home care services, emergency clinics, and other municipal care services (housings for individuals with disabilities, rehabilitation centers, healthcare centers, and psychiatric outpatient clinics).

### Exclusion criteria

Participants who identified as pharmacists, healthcare assistants, or other personnel who had missing data that prevented the identification of their profession were excluded. Participants who identified their profession as ‘*Other*’ without providing further clarification were also excluded. Pharmacists were excluded in the current study because they do not have access to electronic health records system in Norway. Instead, they use the Prescription Intermediary alongside their own digital systems. Furthermore, in Norway, their engagement with patients is not as direct as that of doctors and nurses.

Certain workplaces were considered ineligible: ambulance services, offshore health services, dental clinics, pharmacies, and residential care and service centers. Additionally, participants employed in private hospitals were excluded in the study.

### Data analysis

**Fig. 1:**
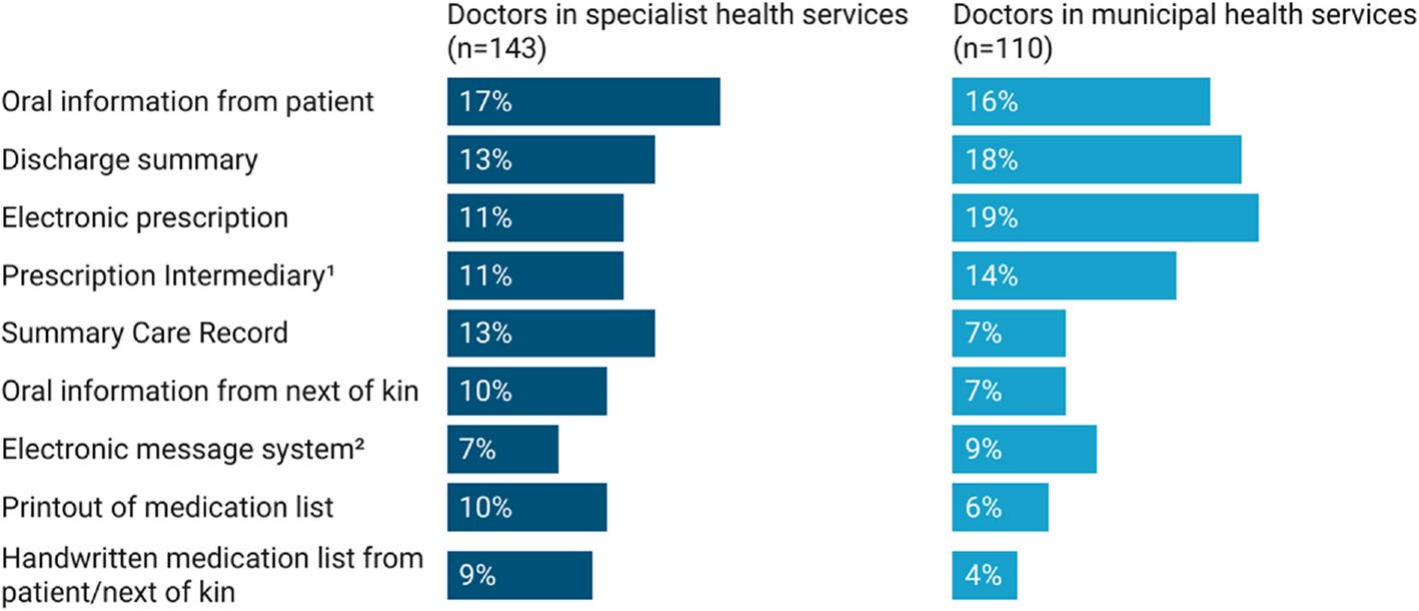
Differences between use of medication information sources between hospitals and municipal settings by doctors. ^1^ The national database for electronic prescriptions. ^2^ The system for enhancing communication and information exchange between homecare services, GPs, and hospitals

**Fig. 2:**
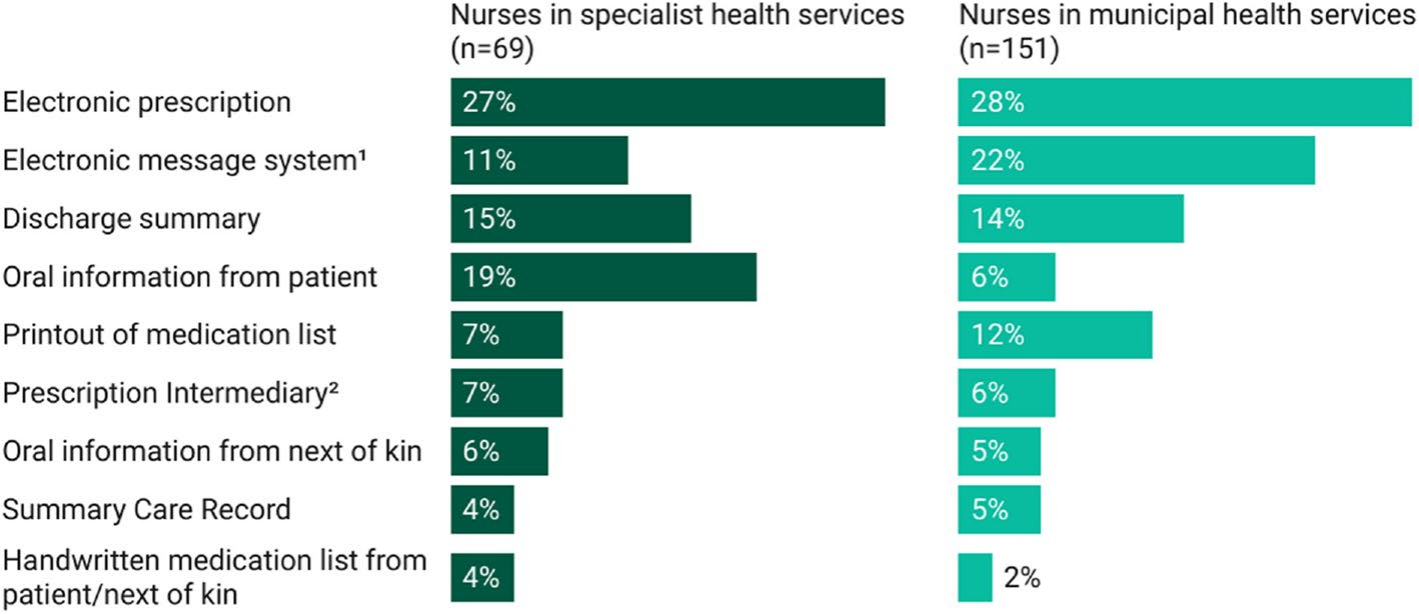
Differences between use of medication information sources between hospitals and municipal settings by nurses. ^1^ The system for enhancing communication and information exchange between homecare services, GPs, and hospitals. ^2^ The national database for electronic prescriptions

Data regarding the management of patients' medication information were analyzed using IBM SPSS Statistics 25 (IBM Corp). Out of the total responses received from doctors and nurses, 14 instances of missing data were identified and excluded from the analysis due to insufficient and/or incomplete responses to address our research questions. Descriptive statistics were used to summarize the demographics of doctor and nurse respondents (Table 1), their use and perceptions of patients’ medication information (Table 2), along with the challenges they perceived in relation to it (Table 3). For the analysis, responses on the 6-point Likert scale were grouped into: "No (scale 1–2, with 1 being *'completely disagree'*)," "Maybe (scale 3–4)," and "Yes (scale 5–6, with 6 being '*completely agree*')." The Pearson Chi-Square test was used to examine univariate associations among categorical variables. Furthermore, data highlighting the sources and frequency of medication information shared between municipal and specialist health services, as reported by doctors and nurses, were analyzed using Microsoft Excel and are depicted in Figs. 1 and 2. These figures showcase the frequency of responses categorized in the survey as “*a few times per week*” and “*daily*”, highlighting the most commonly accessed medication information sources.

**Table 1:** Demographic characteristics of health professionals

	Doctors n (%)	Nurses n (%)	*P*-value^a^
**Age (year)** *N* = 505			< .001
Under 25	2 (0.8)	15 (6.1)	
26–35	102 (39.1)	64 (26.2)	
36–45	88 (33.7)	59 (24.2)	
46–55	47 (18.0)	68 (27.9)	
56–65	20 (7.7)	31 (12.7)	
Over 65	2 (0.8)	7 (2.9)	
**Gender** N = 503			< .001
Female	182 (69.7)	204 (84.3)	
Male	75 (28.7)	37 (15.3)	
Other	4 (1.5)	1 (0.4)	
**Years of professional experience** *N* = 506			.167
0–5	86 (32.8)	58 (23.8)	
6–10	47 (17.9)	47 (19.3)	
11–15	52 (19.8)	47 (19.3)	
16–20	23 (8.8)	33 (13.5)	
21–25	30 (11.5)	28 (11.5)	
≥ 25	24 (9.2)	31 (12.7)	
**Region** *N* = 496			.330
South-and-Eastern Norway	140 (54.1)	147 (62.0)	
Western Norway	54 (20.8)	38 (16.0)	
Central Norway	34 (13.1)	27 (11.4)	
Northern Norway	31 (12.0)	25 (10.5)	
**Workplace** *N* = 473			< .001
Specialist health services			
*Hospital*	143 (56.5)	69 (31.4)	
Municipal health services			
*General practice*	80 (31.6)	0 (0.0)	
*Home care service*	3 (1.2)	95 (43.2)	
*Nursing home*	23 (9.1)	25 (11.4)	
*Emrgency clinic*	2 (0.8)	3 (1.4)	
*Other primary care services*	2 (0.8)	28 (12.7)	
**Municipality size**^b^ *N* = 487			< .001
Large	157 (61.3)	105 (45.5)	
Medium	75 (29.3)	84 (36.4)	
Small	24 (9.4)	42 (18.2)	

^a^*P*-value was computed using chi-square test. *P* < .05 indicates statistical significance

^b^Based on population size: Large > 20000, Medium 5000–20000, Small < 5000. Data source: [25]

**Table 2:** Health professionals’ use and perceptions of patients’ medication information

	Doctors n (%)	Nurses n (%)	*P*-value^a^
Medication list is updated and accurate *N* = 419			
Yes	64 (27.8)	108 (57.1)	< .001
Maybe	115 (50.0)	64 (33.9)	
No	51 (22.2)	17 (9.0)	
Clear responsibility for updating and organizing medication information *N* = 399			.004
Yes	95 (43.0)	106 (59.6)	
Maybe	74 (33.5)	39 (21.9)	
No	52 (23.5)	33 (18.5)	
Easy to obtain specific medication information on prescription from pharmacies *N* = 363			< .001
Yes	116 (52.3)	48 (34.0)	
Maybe	58 (26.1)	30 (21.3)	
No	48 (21.6)	63 (44.7)	
Effective transfer of medication information between my workplace and other healthcare actors *N* = 378			< .001
Yes	32 (15.2)	56 (33.5)	
Maybe	95 (45.0)	66 (39.5)	
No	84 (39.8)	45 (26.9)	
Computer systems offer me good support for medication decision-making *N* = 388			.032
Yes	45 (20.5)	54 (32.0)	
Maybe	92 (42.0)	65 (38.5)	
No	82 (37.4)	50 (29.6)	
Medication handling is safe for my patients *N* = 391			< .001
Yes	60 (27.6)	101 (58.0)	
Maybe	119 (54.8)	59 (33.9)	
No	38 (17.5)	14 (8.0)	

^a^*P*-value was computed using chi-square test. *P* < .05 indicates statistical significance

**Table 3:** Health professionals’ perceived obstacles with patients’ medication information

	Doctors n (%)	Nurses n (%)	*P-*value^a^
Medication lists often contain incomplete prescribing information *N* = 412			< .001
Yes	114 (50.0)	38 (20.7)	
Maybe	73 (32.0)	69 (37.5)	
No	41 (18.0)	77 (41.8)	
Medication lists often contain outdated medication and incorrect dosages *N* = 403			< .001
Yes	128 (56.9)	35 (19.7)	
Maybe	65 (28.9)	62 (34.6)	
No	32 (14.2)	81 (45.5)	
Obtaining an overview of patient medication use is time-consuming *N* = 396			< .001
Yes	164 (73.5)	66 (38.2)	
Maybe	35 (15.7)	51 (29.5)	
No	24 (10.8)	56 (32.4)	
I often have to deal with multiple sources of medication information for same patient *N* = 399			< .001
Yes	186 (84.2)	62 (34.8)	
Maybe	28 (12.7)	50 (28.1)	
No	7 (3.2)	66 (37.1)	
Uncertainty in medication information accuracy *N* = 404			< .001
Yes	116 (52.3)	38 (20.9)	
Maybe	73 (32.9)	66 (36.3)	
No	33 (14.9)	78 (42.9)	
I feel an excessive sense of responsibility for the medication usage of patients *N* = 385			.471
Yes	85 (38.7)	63 (36.8)	
Maybe	70 (32.7)	51 (29.8)	
No	59 (27.6)	57 (33.3)	
I feel a need for the shared medication list *N* = 372			.800
Yes	178 (84.4)	132 (82.0)	
Maybe	26 (12.3)	22 (13.7)	
No	7 (3.3)	7 (4.3)	

^a^*P*-value was computed using chi-square test. *P* < .05 indicates statistical significance

For the qualitative data obtained from open-ended questions, a conceptual content analysis [26] was applied using deductive approach. The respondents were given the option to provide additional information regarding obstacles in medication information transfer and the introduction of shared medication lists in healthcare practice. Participation in the open-ended questions was not mandatory. The content of comments was analyzed by two researchers (BW and USM) using Microsoft Excel. Comments lacking sufficient information for categorization were excluded from the analysis. The two researchers then further discussed and refined categories until a consensus was reached.

## Results

A total of 262 (51.7%) doctors and 244 (48.3%) nurses responded to the survey. Respondents of all age groups answered the survey (Table 1). Survey responses were higher from doctors (72.8%, 190/261) and nurses (78.3%, 191/244) aged 26–55 years. Female respondents were predominant in both professional groups.

The region of South-and-Eastern Norway, which stands as the most populous region in the country, had the highest number of respondents among both professional groups. A substantial proportion of respondents, particularly doctors (90.6%, 232/256, *P* < *0.001*), reported their practice locations to be medium to large-sized municipalities. Among doctors, the main practice settings were hospitals (56.5%, 143/253) and general practice (31.6%, 80/253), whereas nurses were primarily employed in municipal health services (67.3%, 148/220).

### Frequently used sources for patients’ medication information

Doctors in hospital predominantly relied on verbal information directly provided by patients (17%, 24/143) in their regular practice, whereas general practitioners (GPs) depended more on electronic prescriptions (19%, 21/110) and discharge summaries (18%, 20/110) (Fig. 1).

In both specialist (27%, 18/69) and municipal health settings (28%, 42/151), nurses relied on electronic prescriptions to access patients’ medication information (Fig. 2). Those within municipal health settings also indicated frequent use of the electronic message system (22%, 33/151).

### Use and perceptions of patients’ medication information

Doctors reported a significant lower percentage than nurses in terms of medication lists being correct and up to date (27.8%, 64/230), confidence in the safety of their followed medication handling procedures (27.6%, 60/217), as well as having a clear responsibility for maintaining their patients’ medication information (43.0%, 95/221) (Table 2). A considerably higher percentage of doctors (84.8%, 179/211) than nurses (66.4%, 111/167) held negative views when transferring patients’ medication information with other healthcare actors.

### Perceived obstacles with patients’ medication information

Doctors reported a considerably higher proportion of challenges than nurses including encountering incomplete (50%, 114/228), outdated and incorrect medication information (56.9%, 128/225), and harboring uncertainty about the accuracy of medication information they obtained (52.3%, 116/222) (Table 3). In addition, more doctors found obtaining patients’ medication information to be time-consuming (73.5%, 164/223), often requiring them to navigate multiple sources for the same patient (84.2%, 186/221). Both doctors (84.4%, 178/211) and nurses (82.0%, 132/161) expressed the need for a shared medication list.

### Qualitative results from free-text responses

We examined free-text description provided by doctors and nurses and identified obstacles related to transferring medication information (Table 4) and factors necessary for the successfully implementation of a shared medication list (SML) (Table 5). A total of 156 doctors and 81 nurses contributed their perspectives on *obstacles in medication information transfer*, while 124 doctors and 47 nurses shared their insights on *the factors required for introducing SML.*

**Table 4:** Reported obstacles in transferring patients’ medication information

Category	Respondents, n (%)	
	Doctors	Nurses
**Patient groups**		
Multidose dispensing patients	55 (35.3)	6 (7.4)
Patients with cognitive impairments	10 (6.4)	6 (7.4)
Home-dwelling elderly	14 (9.0)	2 (2.5)
Patients with acute illnesses	11 (7.1)	5 (6.2)
Patient with self-management difficulties^a^	18 (11.5)	2 (2.5)
Child and adolescent patients	3 (1.9)	1 (1.2)
**Medication management**		
Slow/inaccurate update of medication list	23 (14.7)	16 (19.8)
Errors/omissions in discharge summary	16 (10.3)	12 (14.8)
Technical difficulties	7 (4.5)	NA
Fail in communication	NA	3 (3.7)
**Transition of care**		
Vertical care^b^	43 (27.6)	26 (32.1)
Horizontal care^c^	14 (9.0)	3 (3.7)

Total number of obstacles by type (doctor *n* = 214, nurse *n* = 82) exceed total number of respondents reporting obstacles (doctor *n* = 156, nurse *n* = 81) because some respondents reported more than one type of obstacle. Frequencies were calculated using 156 (doctor) and 81 (nurse) as a denominator

^a^Patients who struggle to remember or understand their medication lists

^b^Transition of care between different levels of healthcare

^c^Transition of care within the same level of care but across different locations or healthcare settings

**Table 5:** Factors deemed important for implementing shared medication list

Category	Respondents, n (%)	
	Doctors	Nurses
Clear responsibility to update and organize medication information	45 (36.3)	14 (29.8)
Coordinate with existing systems and healthcare actors	39 (31.5)	4 (8.5)
Intuitive, secure login, streamlined	16 (12.9)	9 (19.1)
Accurate, automated medication lists	17 (13.7)	2 (4.3)
A monitoring, quality control, alert system	10 (8.1)	15 (31.9)
Training and incentives for general practitioners	8 (6.5)	1 (2.1)
Allow patients access their medication lists	2 (1.6)	NA
Electronic multidose management platform	4 (3.2)	NA

The total number of factors identified by doctors (*n* = 141) exceeds the total number of doctors (*n* = 124) reporting in this free-text question because some responses were associated with multiple factors. Frequencies were calculated using 124 doctors and 47 nurses as the denominator

### Obstacles in transferring patients’ medication information

Both professional groups encountered similar obstacles when transferring medication information (Table 4). Among all patients, those who had chronic conditions and required complex medication regimes or multidose drug dispensing were often identified as a challenging group by most doctors and nurses. One doctor (#813) expressed, “with patients taking 20 or more medications, reaching a consensus can easily consume over 45 min, which is impractical given the demands of a busy workday.” These challenges became particularly prominent when patients faced difficulties in self-management due to factors such as advanced age or cognitive dysfunction, as they struggled to recall or comprehend their medication lists. As one doctor (#90) stated, “obtaining a comprehensive understanding of patients' medication usage is generally time-consuming and sometimes challenging because patients themselves lack clarity. I find that a significant portion of my working day is dedicated to reconciling medication information.”

The challenges related to medication management frequently identified by most doctors and nurses were primarily associated with slow, delayed, or inaccurate updates of medication lists. These issues were particularly problematic when multiple healthcare providers were involved in a patient’s treatment, especially those with complex medication regimens. According to the doctor (#443), “[if the] summary care record is not updated, it becomes nearly impossible to create an accurate medication list at the hospital without contacting the patient's GP or nursing home.” On the other hand, some GPs expressed their concerns about “not being informed when the medication changes occurred in hospitals (doctor #97)” and “facing difficult in obtaining an overview of prescription changes (doctor #67).” This often resulted in gaps in prescriptions and updates that “placed an additional burden on overworked GPs,” as mentioned by a nurse (#579). The same nurse further emphasized that, “if a locum was involved and unfamiliar with the patient, they would have to write prescriptions for someone they had never met, which is not acceptable.” To obtain accurate medication information that were not yet updated or included in the Prescription Intermediary, “[doctors] have to fax medication changes (doctor #99)” or” or making phone calls to retrieve the information if fax is not available (doctor #83).”

One of the most challenging aspects of securing accurate medication information encountered by the majority of doctors and nurses was due to the lack of digital systems to exchange health information between municipal and specialist health services. A doctor (#83) explained that when there was an admission, they had to spend a significant amount of time gathering medication information from various sources, such as the summary care record, previous discharge summaries, referral from GPs (which often lacked updated list), the patient themselves, next of kin, or Prescription Intermediary. To address this challenge, a doctor (#524) suggested, “a shared medication list should be in place between municipalities and the health authority so that you do not have to spend 20 min finding which medicines to use.”

### Factors deemed important for implementing the Shared Medication List

To successfully introduce SML, the importance of clearly defining responsibilities for updating, organizing, and approving medication information has been emphasized by most doctors and nurses. A nurse (#385) raised concerns regarding the determination of responsibility for ensuring the accountability of the medication list, “the municipal health service updates the list based on orders from GPs and specialist health services, which becomes confusing and complex when minor changes occur, such as adding new text or adjusting dosages.” GPs also expressed their concerns about taking on excessive burdens for medication approval. One GP (#67) mentioned, “I do not have the competence or opportunity to approve [medication in the list].” Another GP (#127) stated that the SML should be the foundation for all medication management, making the last prescribing doctor responsible for its accuracy rather than relying solely on the approval of GP. A doctor (#130) believed that “legislation should mandate equal duty to update the SML for all healthcare actors.”

In addition to defining responsibilities, many doctors and nurses have underscored the need for efficient coordination with existing systems and the establishment of a robust monitoring and quality control framework. Several health professionals have shown enthusiasm for the SML, seeing it as “a significant advancement in ensuring patient safety and the well-being of employees involved in medication management (nurse #307).” However, some have also pointed out that although the implementation of SML will be beneficial, “it may not completely address the issue of patients often using medications different from what is prescribed and dispensed (doctor #231).”

## Discussion

Our study uncovered a significant difference in the concerns expressed by doctors and nurses regarding the acquisition of accurate, safe, and up-to-date medication lists for patients. Specifically, a higher percentage of doctors voiced apprehensions regards to polypharmacy patients or transitioning between primary and specialist healthcare services. To obtain an overview of patients’ medication use, doctors in hospitals primarily relied on verbal information provided by their patients, while GPs and nurses often depended on electronic prescriptions. Doctors, in particular GPs, expressed a sense of being overburdened due to an excessive role to coordinate with multiple healthcare actors when reconciling current medication information. The majority voiced support toward implementing a shared medication list, yet this enthusiasm was underpinned by a desire for a more equitable distribution of responsibility in managing patients' medication information, all while ensuring seamless compatibility with existing digital systems.

### Fragmented sources for medication information

Resource fragmentation with the lack of interconnectedness among different digital solutions can yield fragile and inadequate medication management system [9, 12, 13, 27] and impaired communication between health professionals [28, 29]. Consequently, this situation gives rise to instances of medication discrepancies [30–32], as well as adverse drug events and their associated cost [33]. Our findings illuminated this concern by showing the struggle of health professionals in obtaining, sharing, and exchanging patient medication information, even within the same level of care. This challenge was especially pronounced during transitional care phases, as indicated by both respondent doctors and nurses. This observation is consistent with our earlier qualitative study [9]. Problems that arise include the circulation of several versions of a message from different actors about the same patient (e.g., electronic medication records) [33], resulting in disruptions of the information flow [9, 33], thereby yielding unnecessary and repetitive admin that worsen health professionals’ productivity [34].

Moreover, this fragmentation exacerbates the stress and burden on health professionals, compelling some to undertake the role of 'information detectives' while navigating the process of information retrieval [9]. In Norway, an outdated and clunky administrative approach involving fax communication for conveying medication changes still exists between GPs’ offices and pharmacies. The increasing weight of burdensome administrative tasks, including such, are consuming doctors and nurses at the expense of patient care, personal development, and work-life balance [34]. This situation can be particularly draining in understaffed regions that rely on needing the engagement of short-term locum doctors or nurses, a concern underscored by our respondents, particularly among nurses [35, 36], can be further compounded, jeopardizing patient safety and quality of care, and placing significant financial pressures on local municipalities over the long term.

An adequate, aggregated, and effective information system ensures uniformity among health providers reconciliating medication information, minimizing the risk of communication challenges [37, 38]. The current medication exchange model in Norway is well-suited for supporting sequential, one-way processes such as referral letters and discharge summaries [33]. To improve interpersonal communication across sectors, services, and levels of care, as well as safeguarding the quality of information on medication, a paradigm shift that integrates local solutions distributed in hospital, general practices, municipalities, and others becomes crucial. This underscores the significance of the concept of the Shared Medication List, acting as a centralized information hub to foster communication and curtail the occurrence of preventable medication errors due to communication failure [39]. Despite being in its early piloting stage in Norway, the implementation of the SML carries the potential to enhance patient safety and advance the overall quality of healthcare delivery.

### Role clarification in the management of medicine

Clinical roles tend to overlap when the responsibilities of each team member are insufficiently defined [40, 41], often exacerbated by communication gaps across different services and levels of care [42]. Our findings specifically underscored concerns from doctors, particularly GPs, and nurses about ambiguities in managing patients’ medication information. Consequently, both professional groups emphasized the need for clearly defined roles when introducing SML. This aligns with qualitative research findings from our pre-study by Manskow and Kristiansen [9]. In addition, studies by Hammar et al. [39, 43] and Brault et al. [41] resonate with our observations, highlighting health professionals’ call for clear, unambiguous professional role clarification to optimize medication management process and to ensure patient safety.

In Norway, polypharmacy patients are often managed by multiple actors within different organizations and levels of care. In such case, a GP or a home care nurse may clearly be hesitant to discontinue a drug recommended by a specialist, particularly if the patient has been using it over an extended period or if they are no longer followed by the specialist. To mitigate unclear, overlapping responsibilities, proactive measures must be taken upon introducing SML to the local health services. On an organizational level, one practical tip involves implementing training initiatives [39] and fostering trust and mutual understanding among healthcare professionals through interactive sessions [44]. At the professional competence level, establishing regular, brief interprofessional meetings can effectively encourage communication and collaboration among healthcare providers who are involved in the care of the same patient [44–46].

### Patient involvement in medication safety

Patients on multidose dispensing, or polypharmacy, are particularly vulnerable to medication errors [47–49]. Our findings support this notion, with both doctors and nurses identifying polypharmacy as a primary challenge in transferring and managing medication information, and this becomes even more pronounced for elderly patients with cognitive impairments. Surprisingly, only a small number of doctors from our study were supportive of patients having the ability to edit, comment on, or correct their own medication records in the SML. This is concerning, especially in light of recent research from Denmark that identified frequent discrepancies between medication listed in the SML and those patients actually used [50, 51]. With the impending national rollout of the SML in Norway, now comes a unique opportunity to increasingly, actively involve patients, which might have a significant impact to maintain accurate medication lists and reduce discrepancies that arise when physicians fail to promptly update these lists [39]. Encouraging open communication among patients, prescribers, pharmacists, and nursing staff has the potential to further reduce medication errors [47, 52]. Despite promising, patients currently remain unable to offer feedback or modify prescriptions on their actual medication use in countries where the SML has been introduced, such as Denmark, Sweden, Norway, and Finland [39]. To foster a safer medical landscape, forthcoming policies across Nordic countries should lay the groundwork for enhanced patient engagement, assisted by the guidance of their health professionals [53].

### Strengths and limitations

This article adds knowledge to the current conditions for obtaining and sharing medication information within the Norwegian healthcare system, serving as a baseline reference for forthcoming planned research both *during* and *after* the implementation of the Shared Medication List. To our knowledge, this is the first cross-sectional survey study that seeks to understand the experiences and perceived obstacles faced by doctors and nurses in managing patients’ medication information. Besides, our study included a diverse range of respondents from various healthcare professions, settings, and regions throughout Norway, offering a broad demographical perspective regarding this topic.

However, certain limitations should be considered. First, the reliance on self-reported data obtained through the survey might introduce bias. Second, the absence of data on the exact number of unique users visiting the survey means we could not determine the actual response rate. This presents possible risks of nonresponse bias, leaving us uncertain if nonrespondents possess different characteristics, such as age, profession, or workplace, compared to respondents. This could impact the robustness of our data. Moreover, the questionnaire underwent only face validity, which is the weakest type of validity “testing”. We recognize the lack of in-depth statistical validation test, like content and construct validity, as a limitation, since we cannot fully ensure that our questions are measuring what it aims to measure, or the concept that it is intended to measure (i.e., perceived obstacles of managing patients’ medication information). Third, while the free-text responses from health professionals offered some insights, they tended to be brief and may not have fully conveyed the specific details of *why* and *how* the obstacles were encountered and handled during medication management. Hence, in-depth interviews or focus groups could be valuable in future research. Another possible limitation is that most respondent doctors had short professional experience (0–5 years), which may lead to selection bias in the age range. Lastly, the choice to employ a company, Medlytics, for respondent recruitment might introduce another layer of selection bias, as we did not directly control the recruitment or data collection.

## Conclusions

The study explored the experiences and perceived obstacles faced by Norwegian health professionals when obtaining and exchanging medication information within the current digital systems. The findings revealed a prevalent issue among both doctor and nursing staff of resource fragmentation and unclear responsibilities when managing patient medication. Doctors exhibited more uncertainty compared to nurses regarding the accuracy of the medication list. While the introduction of a shared medication list has the support from both professional groups, its successful implementation mandates the establishment of proactive training programs, including clarifying roles and responsibilities among clinical staff.

### Supplementary Information

**Additional file 1:** **Supplementary 1.** STROBE Checklist.

**Additional file 2:** **Supplementary 2.** Survey.

**Additional file 3:** **Supplementary 3.** Which electronic health record (EHR) system do you have in your main workplace in specialist health services.

**Additional file 4:** **Supplementary 4.** Which electronic health record (EHR) system do you have in your main workplace in municipal health services.

## Data Availability

The datasets used and/or analyzed during the current study are available from the corresponding author on reasonable request.

## Abbreviations

SCR: Summary Care Record
SML: Shared Medication List
GP: General practitioner
EHR: Electronic health records

## Footnotes

1 Medlytic is a company helping health care professionals to test new technology before and after it is launched and provide their input on areas that concern them.
