# Correction: Health professionals’ experience and perceived obstacles with managing patients’ medication information in Norway: cross-sectional survey

**DOI:** 10.1186/s12913-024-10967-4

**Published:** 2024-04-23

**Authors:** Bo Wang, Unn Sollid Manskow

**Affiliations:** 1 Norwegian Centre for E-Health Research, University Hospital of North Norway, Sykehusvegen 23, Forskningsparken, Tromsø Norway; 2 Department of Health and Care Sciences, Centre for Care Research, UiT-The Arctic University of Norway, Tromsø, Norway

**Correction to: BMC Health Services Research (2024) 24:68.**

10.1186/s12913-023-10485-9

In this article, the authors identified the following errors:

1. ‘Role clarification in the management of medicine’ subsection: In the sentence beginning “Clinical roles tend to overlap when…”, the duplicated text “across services and levels of care” needed to be removed.
2. ‘Patient involvement in medication safety’ subsection: In the sentence beginning “With the impending national rollout.”, the duplicated text “actively involving patients is necessary to” needed to be removed.
3. References: Reference 1 needed to be corrected from “Organization WH. Medication without harm. World Health Organization; 2017” to “World Health Organization. Global Patient Safety Challenge: Medication Without Harm. World Health Organization; 2017. http://www.who.int/patientsafety/medication-safety/medication-without-harm-brochure/en/. Accessed 13 Jan 2024.”
4. References: Reference 8 needed to be corrected from “omsorgsdepartementet H-o. Meld. St. 28 (2014?2015). Legemiddelmelding 2015 - Riktig bruk - bedre helse. In: Services TRNMoHaC, editor. 2015.” to “Helse-og omsorgsdepartementet. Legemiddelmeldingen ? Riktig bruk ? bedre helse. Meld St. 2014;28(2014-2015)”.
5. References: Reference 14 was presented incorrectly as “!!! INVALID CITATION!!! (14, 15).” As the correct reference information for reference 14 was a duplicate of reference 9 (Manskow and Kristiansen, 2021), it needed to be removed and its citation in the main text needed to be re-ordered (including all subsequent reference citations).
6. References: As reference 26 was a duplicate of reference 9 (Manskow and Kristiansen, 2021), it needed to be removed and its citation in the main text needed to be re-ordered (including all subsequent reference citations).
7. References: Reference 17 needed to be corrected from “(KS) TNAoLaRA. Kommunal sektors ambisjoner på e-helseområdet. Felles plan og rammeverk. ISBN 978–82–93866–09–1; 2022 May 2022.” to “The Norwegian Association of Local and Regional Authorities (KS). The municipal sector’s ambitions in the area of e-health. Joint plan and framework (In English). ISBN 978–82–93866–09–1; 2022 May 2022. https://www.ks.no/fagomrader/digitalisering/felleslosninger/digitalisering-i-helse-og-omsorgsektoren-e-helse/om-e-helse/. Accessed 13 Jan 2024.”
8. References: Reference 18 needed to be corrected from “e-helse Df. Nasjonal E-helsestrategi 2023–2027. Direktoratet for e-helse; 2023. p. 17.” to “The Norwegian Directorate for eHealth, National e-health strategy for the health and care sector (In English). 2023. p. 17. https://www.ehelse.no/strategi/nasjonal-e-helsestrategi-for-helse-og-omsorgssektoren. Accessed 13 Jan 2024.”

The original article has been corrected.

