# Supplementary material for: Health professionals’ experience and perceived obstacles with managing patients’ medication information in Norway: cross-sectional survey
Source: BMC Health Serv Res. 2024 Jan 13;24:68. doi: 10.1186/s12913-023-10485-9 (PMC10790274; doi:10.1186/s12913-023-10485-9)
Supplement: Supplementary file 2 — Additional file 2: Supplementary 2. Survey. [file 12913_2023_10485_MOESM2_ESM.docx]

Supplementary 2. Survey

**Autumn 2022 Shared Medication List Survey for Health Personnel**

Background and purpose of the survey

The questionnaire is aimed at healthcare personnel within the primary healthcare service in Norway who prescribe, administer, or handle medicines. The questionnaire is part of a national research project under the auspices of the Norwegian Center for E-health Research (NSE). NSE will conduct follow-up research on the introduction of the Shared Medication List (SML) in the primary healthcare service in the period 2020-2025.

The purpose of this survey is to examine how medication management works today, and to identify perceived obstacles.

Your identity will be kept hidden. When hidden identity is used in surveys, no identifiable information, such as browser type and version, IP address, operating system, or email address, be saved with the response. This is to protect the respondent's identity.

The first 7 questions are background information about you.

1) * Which profession do you belong to?

| 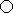 Doctor 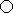 Nurse 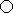 Social educator 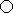 Pharmacist 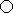 Healthcare worker  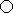 Other, please describe: |
| --- |

2) Your age

| 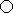 Under 25 years |
| --- |
| 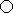 25-35 |
| 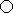 36-45 |
| 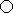 46-55 |
| 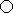 56-65 |
| 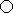 Over 65 years |
| 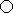 Prefers not to provide |

3) * Years of experience in the profession

| 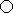 0-5 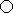 6-10 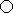 11-15 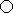 16-20 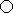 21-25 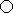 More than 25 years |
| --- |

4) * Gender

| 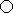 Female 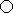 Male 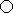 Other 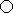 Prefers not to provide |
| --- |

5) * Which county do you work in?

| 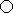 Troms og Finnmark |
| --- |
| 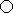 Nordland |
| 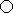 Trøndelag |
| 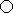 Møre og Romsdal |
| 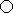 Innlandet |
| 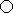 Vestland |
| 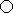 Viken |
| 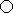 Oslo |
| 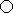 Vestfold og Telemark |
| 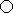 Rogaland |
| Agder 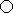 |

6) Do you work in a small, medium-sized, or large municipality? (Small: < 5,000 residents, medium-sized: 5,000-20,000, large: > 20,000 residents)


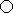
 Liten
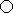
 Mellomstor
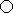
 Stor

7) * Your primary workplace:

| Nursing home |
| --- |
| Home care service |
| Emergency clinics  Pharmacy  Hospital pharmacy  Care and respite residence |
| GP office |
| Hospital |
|  |
| Other, please describe: |

**8) * Which electronic health record system do you have at your workplace?** ***(free-text)***

Healthcare personnel and pharmacy staff use many different information sources to obtain an overview of the medications a patient is being treated with. Please specify the different types you use in your work and indicate how often you use these sources.

9) * Specify which information sources you use, and how often, to determine the medications a patient is being treated with:

|  | Never | Sometimes a year | Sometimes per month | Sometimes per week | Daily | Not relevant |
| --- | --- | --- | --- | --- | --- | --- |
| Electronic prescription |  |  |  |  |  |  |
| Prescription Intermediary |  |  |  |  |  |  |
| Summary Care Record |  |  |  |  |  |  |
| Oral information from patient |  |  |  |  |  |  |
| Oral information from next of kin |  |  |  |  |  |  |
| Handwritten medication list from patient/next of kin |  |  |  |  |  |  |
|  |  |  |  |  |  |  |
| Printout of medication list from patient (received from pharmacy, doctor, home care service) |  |  |  |  |  |  |
|  |  |  |  |  |  |  |
| Electronic message system |  |  |  |  |  |  |
| Discharge summary |  |  |  |  |  |  |

**Questions about handling patients’ medication information**

*When we refer to the medication list, we mean the list of a patient's current medications that you primarily use today, such as the medication list in your electronic health record system or printed from another system.*

Rate on a scale from 1-6 (1=strongly disagree, 6=strongly agree) to what extent you agree/disagree with the statements:

10) I trust that the medication list I primarily use is up-to-date and accurate.

| 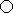 1 Strongly disagree 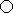 2 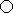 3 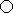 4 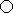 5 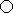 6 Strongly agree 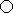 7 Not applicable/don’t know |
| --- |

11) It is common for the medication list to lack information about which medications a patient has been prescribed.

| 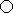 1 Strongly disagree 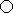 2 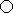 3 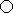 4 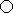 5 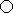 6 Strongly agree 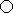 7 Not applicable/don’t know | 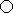 1 Helt uenig 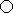 2 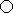 3 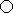 4 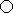 5 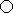 6 Helt enig 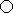 Vet ikke |
| --- | --- |

12) It is common for the medication list to contain outdated medications or incorrect dosages.

| 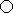 1 Strongly disagree 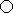 2 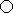 3 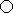 4 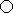 5 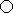 6 Strongly agree 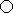 7 Not applicable/don’t know | 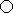 1 Helt uenig 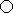 2 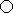 3 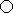 4 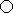 5 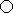 6 Helt enig 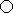 Vet ikke |
| --- | --- |

13) The responsibility to update and maintain the information in the medication list is clear to me.

| 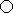 1 Strongly disagree 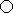 2 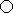 3 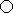 4 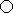 5 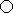 6 Strongly agree 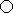 7 Not applicable/don’t know | 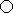 1 Helt uenig 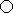 2 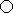 3 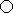 4 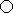 5 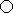 6 Helt enig 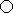 Vet ikke |
| --- | --- |

14) It is easy to obtain information about which medications a patient has picked up with a prescription from a pharmacy.

| 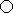 1 Strongly disagree 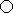 2 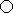 3 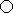 4 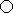 5 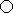 6 Strongly agree 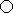 7 Not applicable/don’t know | 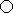 1 Helt uenig 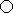 2 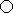 3 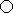 4 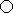 5 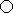 6 Helt enig 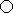 Vet ikke |
| --- | --- |

15) It is unnecessarily time-consuming to obtain an overview of which medications a patient is using.

| 1 Strongly disagree 2 3 4 5 6 Strongly agree 7 Not applicable/don’t know | 1 Helt uenig 2 3 4 5 6 Helt enig Vet ikke |
| --- | --- |

16) I must often deal with multiple sources of information about the current medications for the same patient.

| 1 Strongly disagree 2 3 4 5 6 Strongly agree 7 Not applicable/don’t know | 1 Helt uenig 2 3 4 5 6 Helt enig Vet ikke |
| --- | --- |

17) I often feel uncertain about whether the information I have about which medications the patient is using is correct.

| 1 Strongly disagree 2 3 4 5 6 Strongly agree 7 Not applicable/don’t know | 1 Helt uenig 2 3 4 5 6 Helt enig Vet ikke |
| --- | --- |

18) I mainly experience that the management of medications is safe for the patients I encounter in my work.

| 1 Strongly disagree 2 3 4 5 6 Strongly agree 7 Not applicable/don’t know | 1 Helt uenig 2 3 4 5 6 Helt enig Vet ikke |
| --- | --- |

19) The transfer of information about patients' current medications works well between my workplace and other actors (such as between primary and specialist healthcare, within primary healthcare).

| 1 Strongly disagree 2 3 4 5 6 Strongly agree 7 Not applicable/don’t know | 1 Helt uenig 2 3 4 5 6 Helt enig Vet ikke |
| --- | --- |

20) * I feel like I take on more responsibility than I should in my role regarding patients' medication use.

| 1 Strongly disagree 2 3 4 5 6 Strongly agree 7 Not applicable/don’t know | 1 Helt uenig 2 3 4 5 6 Helt enig Vet ikke |
| --- | --- |

21) Are there patient groups, situations, or transitions where information transfer about patients' medications works less well/effectively today? Please describe or provide examples:

23) The data systems I use provide me with good support for making decisions about medications.

| 1 Strongly disagree 2 3 4 5 6 Strongly agree 7 Not applicable/don’t know | 1 Helt uenig 2 3 4 5 6 Helt enig Vet ikke |
| --- | --- |

Shared Medication List

Shared Medication List (SML) is a new digital solution that will be implemented nationally across the entire healthcare system in the coming years. SML aims to provide access to a patient's medication information in one unified, national overview in real-time, regardless of where one works within the healthcare system and which digital systems are used. SML is designed to give healthcare professionals access to updated information about a patient's medication use and focuses on facilitating the sharing of updated medication information across various healthcare entities (hospitals, GP offices, municipal health and care services, etc.).

24) I perceive a need for the Shared Medication List (as described above).

1 Strongly disagree 2 3 4 5 6 Strongly agree 7 Not applicable/don’t know

25) Do you see any obstacles or issues related to the implementation of the Shared Medication List? *(Free-text)*

|  |  |  |  |  |  |  |
| --- | --- | --- | --- | --- | --- | --- |
|  |  |  |  |  |  |  |
|  |  |  |  |  |  |  |
|  |  |  |  |  |  |  |
|  |  |  |  |  |  |  |
|  |  |  |  |  |  |  |
|  |  |  |  |  |  |  |
|  |  |  |  |  |  |  |

100 % completed

Progress:

Created with [Questback Experience Management](https://www.questback.com/no/)

Free trial subscription – [create a survey](https://www.questback.com/lp/no/signup/?topic=quest) with Questback
