# Supplementary material for: Health professionals’ experience and perceived obstacles with managing patients’ medication information in Norway: cross-sectional survey
Source: BMC Health Serv Res. 2024 Jan 13;24:68. doi: 10.1186/s12913-023-10485-9 (PMC10790274; doi:10.1186/s12913-023-10485-9)

Supplementary 4. Which electronic health record (EHR) system do you have in your main workplace in municipal health services


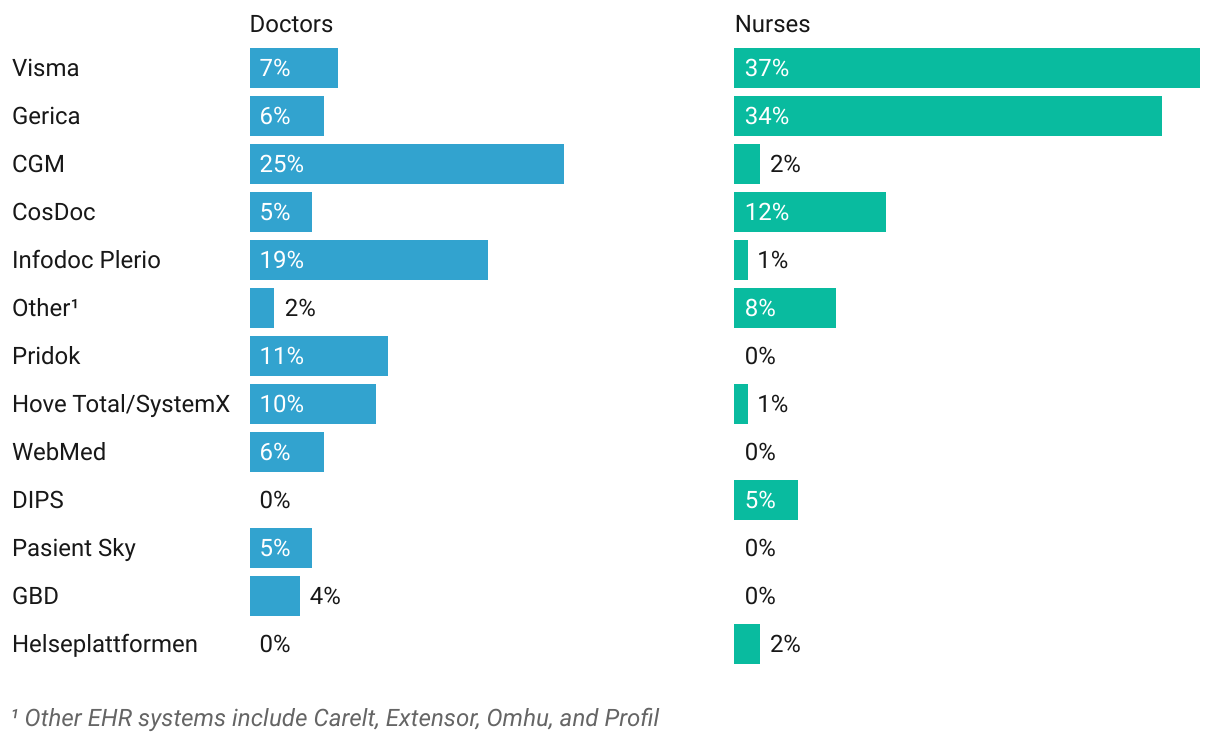

Supplement: Supplementary file 4 — Additional file 4: Supplementary 4. Which electronic health record (EHR) system do you have in your main workplace in municipal health services. [file 12913_2023_10485_MOESM4_ESM.docx]
